# The Characteristics of Whey Protein and Blueberry Juice Mixed Fermentation Gels Formed by Lactic Acid Bacteria

**DOI:** 10.3390/gels9070565

**Published:** 2023-07-11

**Authors:** Wenqiong Wang, Yuxian Wang, Xian Liu, Qian Yu

**Affiliations:** 1 College of Food Science and Engineering, Yangzhou University, Yangzhou 225127, China; wenqiong@yzu.edu.cn (W.W.); wangyuxian0708@163.com (Y.W.); liuqian7734@126.com (X.L.); 2 Weiwei Food & Beverage Co., Ltd., Xuzhou 221114, China; 3 Jiangsu Key Laboratory of Dairy Biotechnology and Safety Control, Yangzhou University, Yangzhou 225127, China

**Keywords:** blueberries, whey protein, lactic acid bacteria, fermentation gels

## Abstract

The properties of blueberry juice and whey protein gels formed by the mixed fermentation of *L. plantarum* 67 and *L. paracasei* W125 were investigated. The state of the gels, including the colour and surface morphology of the microspheres, showed significant changes with different fermentation times. The polyphenolic, flavonoid, and protein release of whey protein or combined blueberry juice fermented gels under in vitro digestion were investigated. The whey protein and blueberry juice fermented gels had more small pores, with a honeycomb structure, compared to whey protein fermented gels. The hardness of the gels was increased after fermentation for 7 h for the whey protein gels and whey protein mixture blueberry juice gels. The storage modulus and water-holding capacity of the gels were increased between fermentation times of 6 h and 8 h. The swelling rates of the whey protein gels fermented for 7 h and whey protein mixed blueberry juice gels fermented for 8 h and kept in pepsin-free simulated gastric fluid for 1 h had higher values. The release of polyphenols, flavonoids, and protein for the fermented gels was higher at fermentation of 7 h in the in vitro digestion experiment. We found that the chewiness of the whey protein gels, or whey protein mixed fermentation gels, was higher at a fermentation time of 7.5 h and 8 h. However, the cohesiveness values were not significantly different. Therefore, whey protein fermented gels and whey protein mixed blueberry juice fermented gels should be fermented for more than 7 h. This facilitates the release of polyphenols, flavonoids, and protein in the gastric juices.

## 1. Introduction

Whey protein concentrate has gelation properties, and has therefore been used as an ingredient to improve the organisation, flavour, and texture of food products [1]. Gelation of proteins usually occurs by thermal denaturation or acid induction. Acid-induced gel formation requires a heating process for protein unfolding and even denaturing of the protein structure, which exposes more hydrophobic amino acids to the protein surface. This creates more protein aggregates [2]. Subsequently, a gradual decrease in pH leads to charging of the protein, changing the balance between electro static repulsion and van der Waals interactions [3], and favouring interactions through hydrophobic forces. The process of protein gelation is always based on the interaction between proteins, which may be due to the interaction between electrostatic forces, hydrogen bonds or hydrophobic forces, or covalent bonds, such as disulfide bonds [4]. The protein network is a highly ordered three-dimensional network structure or matrix [5]. In this equilibrium system, if attraction is dominant, water will be discharged from the gel matrix to form condensation. If repulsive forces dominate, it is difficult to form a network structure. Therefore, the structure and properties of protein molecules can affect the formation of the gel. Different structures of protein aggregation act as a precursor to gel formation, determine the form structure and rheological properties of the gel, and often, in a covalent bond formation of the gel structure, are reversible, while hydrophobic interactions and disulfide bond formation of the gel structure are not reversible [6]. Today, cold gels can be induced by the addition of salts, such as calcium ions, which can form cold gels at room temperature [7,8,9], or by the addition of a slow-release acidifying agent such as gluconolactone-δ-lactone (GDL), which is used to lower the pH and thus induce cold gel formation [10,11]. In the process of acid-induced whey protein cold gel formation, thermal induction first induces the denaturation of the whey protein, which exposes some hydrophobic groups or regions within the molecule to the molecular surface [2]. During the acidification process, when the pH value is close to the isoelectric point of the whey protein, the electrostatic charge of the protein molecule is decreased. Thus, the protein molecules approach each other. Then, the denatured whey protein aggregates form the initial acid-induced protein network structure by physical force [12]. The sulfhydryl groups exposed on the protein surface then form disulfide bonds between the protein molecules, either by their own oxidation or by conversion between sulfhydryl–disulfide bonds (the latter being dominant), which in turn form stable whey protein cold gels by intermolecular cross-linking [4].

Whey protein is usually used as the main component of gels, due to the denatured protein forming a net structure or connecting with other molecules such as polysaccharides and polyphenols [13]. Research has mainly focused on the use of amino acids, polysaccharides, salts, and other substances to form the entrainment of whey protein gels and their effects on the characteristics of whey protein gels. Research on the mechanism of whey protein gel solution principles of gels with lactic acid bacteria fermentation and experimental studies of the properties of these gels is relatively limited. At present, most whey protein gels are applied in food, aiming to affect the texture, quality, and taste of food, and gels formed by whey protein can also be used as food embedding or carrier materials. There are relatively few studies on the application of lactic acid bacteria fermentation gels in other applications.

**Figure 1:**
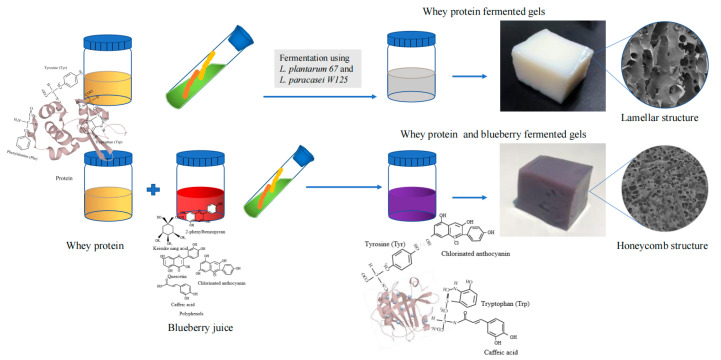
Schematic diagram showing the preparation of whey protein fermented gels or whey protein and blueberry fermented gels.

Lactic acid is formed in the fermentation process to reduce the pH value in the system, which is conducive to the formation of cold gels, causing the protein to form a relatively stable network structure which can better lock the water and nutrients into the system. Blueberry juice and whey protein fermentation products were shown to protect probiotic cells during in vitro gastrointestinal experiments in a previous study. The bioactive components of anthocyanins were also protected during in vitro digestion. It is of significance to proceed with the development of functional jelly foods or fermented food gels with chemical products with living bacteria. In this experiment, a blueberry and whey protein concentrate was fermented by lactic acid bacteria, as shown in Figure 1. We assessed the gel characteristics at different fermentation times under mixed strain fermentation. The changes in gel colour and texture at different fermentation times were observed. Analysis of the internal and external structure of the gels through macroscopic and microscopic observations, and analysis of the network structure, were also demonstrated via the analysis of water holding capacity, swelling rate, and in vitro digestion. Textural and rheological analysis of the gels provided a better understanding of the gel properties of different gel samples. By combining several properties, the quality of the gels could be judged.

## 2. Results and Discussion

### 2.1. Macrostructure Observation

**Figure 2:**
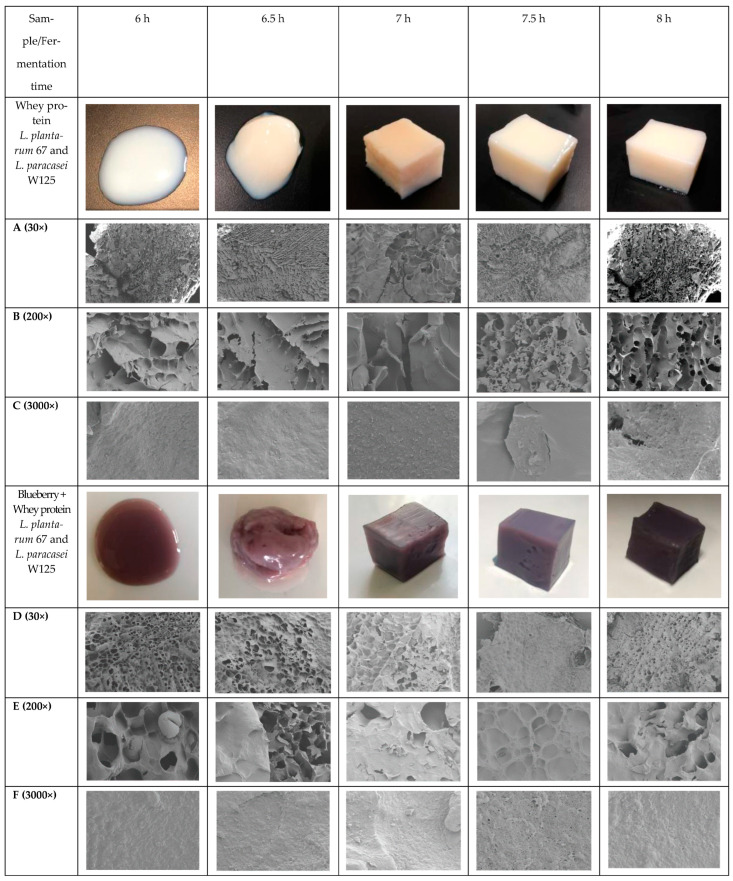
Surface morphological changes of whey protein gels formed by *L. plantarum* 67 and *L. paracasei* W125 with different fermentation times by the magnification of A (30×) B (200×) and C (3000×). Whey protein mixture blueberry juice gels formed by *L. plantarum* 67 and *L. paracasei* W125 with different fermentation times by the magnification of D (30×) E (200×) and F (3000×).

As shown in Figure 2, the gels were gradually formed with increased fermentation time. The fluidity of whey protein or protein mixed blueberry juice fermentation gels was decreased after fermenting for 6.5 h, and the hardness and elasticity were increased. However, there was no significant difference on the surface at a fermentation time of 7 h to 8 h. The whey protein system inoculated with *L. plantarum* 67 and *L. paracasei* W125 was an off-white colour, as shown in Figure 2. The state of the whey protein fermentation system was liquid at a fermentation time of 6 h, with less elasticity. At a fermentation time of 6.5 h, the whey protein fermentation system began to show gelation, with a higher viscosity, which did not take shape and could easily show soft collapse. At a fermentation time of 7 h, the whey protein fermentation gel was formed, without liquid leaking out. Fermentation for 7.5 h and 8 h led to the whey protein gel still showing an off-white colour, without liquid leaking. The state of the blueberry juice and whey protein mixed system inoculated with *L. plantarum* 67 and *L. paracasei* W125 at 6 h was still liquid. The sample was a purple-grey colour. Like the whey protein gels, the whey protein and blueberry juice fermentation gel formation was still occurring from 7 h to 8 h. In order to analyse the structural changes during the fermentation process, SEM was used to observe the microstructure of the whey protein or blueberry juice gels. As shown in Figure 2, the structure of the whey protein fermentation gels was smoother than the whey protein and blueberry juice mixture fermentation gels. There were more pores in the whey protein and blueberry juice fermentation gels. When protein molecules are positively or negatively charged under acidic or alkaline conditions, they tend to form an ordered mesoporous network structure [14]. The whey protein fermentation gels developed an increasingly scaly and rough structure as the fermentation time increased. This may have been related to the gel forming process, in which more acid products in the system electrify the whey protein, forming a charge repulsive effect. This was beneficial to the formation of the gel stereoscopic network structure. However, there were many small pores and a uniform distribution, which may have been due to the phenolic or anthocyanin contact with the whey protein forming a space steric hindrance during the fermentation time. The interior structure of the pores was smooth. The microstructure of the whey protein and blueberry juice was relatively rough compared to the whey protein gels, based on the 3000× image results.

### 2.2. Colour Analysis

Table 1 shows the colour changes of *L. plantarum* 67 and *L. paracasei* W125 fermented gels at each fermentation time. The lightness was increased for whey protein fermentation gels at 6.5 h. The changes in lightness were not significant from 7 h to 8 h for whey protein fermentation gels. However, the lightness of the whey protein and blueberry juice fermentation gels was significantly higher than the whey protein gels (*p* < 0.05). The value of Δa for whey protein and blueberry juice fermentation gels was significantly higher than for whey protein gels, which indicated that the redness was increased. The difference in redness of the whey protein and blueberry gels at a fermentation time of 6 h to 8 h was not significant. This was related to the colour of anthocyanins in the blueberry juice, which changed with different pH conditions [15]. The basic structure of anthocyanins is a glycosylated polyhydroxyl or polymethoxy derivative of 2-phenylbenzopyrane, usually with a molecular weight between 400 and 1200 (medium sized biomolecules) and containing two benzyl rings, A and B. Anthocyanins usually contain a single glycoside unit, but most anthocyanins contain two, three, or more sugars attached to multiple locations or present as oligosaccharide side chains. The concentration and colour type of anthocyanins are influenced by hydroxyl and methoxy bases: if more hydroxyl is present, the colour becomes bluer. If more methoxy is present, it is redder [16]. The colour of anthocyanins varies with pH. The colour was red when the pH < 7. The colour was purple when the pH was 7–8. The colour was blue when pH > 11. Delphinidin and petunidin were red under acidic pH conditions. The yellowness of whey protein gel changes were not significant from a fermentation time of 7 h to 8 h. The blueness of whey protein and blueberry juice gels was slightly increased as fermentation time increased. The yellowness of whey protein gels was higher than whey protein and blueberry juice fermentation gels. This indicated that whey protein or whey protein and blueberry juice gels, once formed, do not undergo significant colour changes that can be detected by the human eye.

### 2.3. Water Holding Capacity and Swelling of the Fermentation Gels

**Figure 3:**
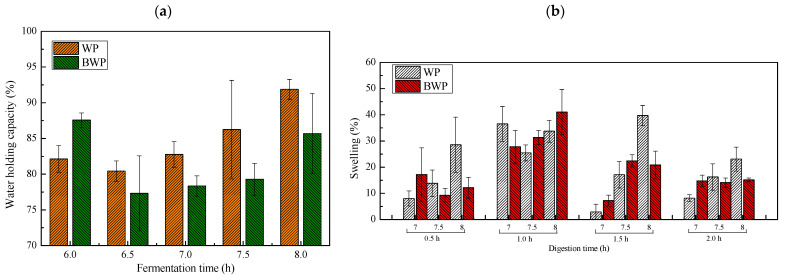
Water holding capacity (**a**) and swelling ratio (**b**) of whey protein mixture blueberry juice gels formed by *L. plantarum 67* and *L. paracasei* W125 fermentations for different lengths of time.

Water holding capacity is an important standard to evaluate the quality of a gel, which determines the function of the gel in technical applications and storage. The water retention ability of gels is related to their microstructure, including the size of gel pores, surface roughness, and the type of protein in the gel. The microporous structure of the gel holds the water molecules tightly in the gel network through capillary forces [17]. Figure 3a shows the water holding capacity changes of the gels at different fermentation times. The gels exhibited the strongest ability to hold water following fermentation for 8 h in the case of whey protein gels. This was because, with the increase in fermentation time, the network structure of the gel became tighter and it was able to bind more water, so the water holding capacity was higher. The gel absorbs water, and this can cause the gel to swell. This is also due to the non-reacted carboxylic acid groups in the structure of crosslinked proteins. Microbial fermentation produces acid, which promotes the aggregation of whey proteins to form a gel structure, and protein aggregation forming a network structure increases its retention ability for water molecules, which is related to the unreacted carboxyl groups in the protein in the gel structure.

However, the WHC of whey protein and blueberry juice fermentation gels was higher than whey protein gel that was fermented for 6 h. The capacity was lower than whey protein gels following fermentation for 6.5 h to 8 h. This was due to the free hydroxyl content from the blueberry being higher following fermentation for 6 h, which is strongly hydrophilic. The hydroxyl content was decreased as fermentation time increased, which led to contact of the whey protein molecular surface by hydrogen bonds [15]. Therefore, the water holding capacity was decreased. Swelling is an important parameter controlling the release rate of cargos from hydrogels [17]. Figure 3b shows the changes of samples at different fermentation times in terms of swelling rate at different times under the action of gastric fluid. For the fermentation gels, the swelling rate showed an increase at 1.5 h in gastric fluid and then decreased at 2 h and 2.5 h. This protein gel network structure absorbs water and bulges very quickly under gastric fluid exposure for 1.5 h. At an ambient pH of 2, acidic protein molecules become positively charged. The electrostatic repulsion forces the protein molecules to push away from each other, thus increasing the gel’s dilatancy [14]. The swell rate was decreased following exposure to gastric solution for 1.5 h and 2 h. This was related to the gels being broken, or fewer water molecules entering the network structure, finally moving them to equilibrium.

### 2.4. The Polyphenolic, Flavonoid, and Protein Release of Whey Protein or Combined Blueberry Juice Fermentation Gels

**Figure 4:**
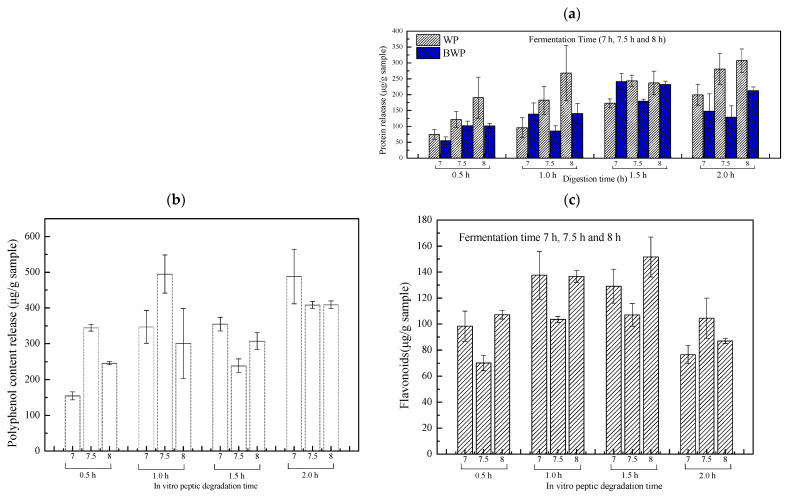
The release of protein (**a**), polyphenols (**b**), and flavonoids (**c**) from whey protein mixture blueberry juice gels formed by *L. plantarum 67* and *L. paracasei* W125 fermentation following different times of in vitro peptic degradation.

As shown in Figure 4a, the protein release from the whey protein system inoculated with 67 + W125 at different digestion times increased with increasing fermentation time. As the retention time in the simulated gastric juice increased, the action of the gastric juice led to a loosening of the gel matrix structure, indicating an increased disruption of the gel system by the gastric juice and the appearance of more pore-like rough structures in the gel system. This was mainly related to the disruption of the binding between the whey proteins to each other, and between the proteins and the polyphenols, resulting in an increase in the contact area of the gel with the water in the gastric juice or an increase in the interactions with the water molecules. The simulated gastric juice eroded the surface and transported water into the gel, while acids and enzymes caused the release of the proteins inside the gel [18]. Similarly, in the blueberry-whey protein mixed system of 67 + W125 at different digestion times, the protein release increased with increasing fermentation time from 0.5 to 1.5 h, mainly due to the increase in the gastric acid destruction time, the disruption of the binding of substances in the blueberry to the protein, the disintegration of the protein aggregates, and the release of the proteins. It started to decrease at 1.5–2 h, possibly as the proteins reached their maximum value, when they started to bind to free substances, forming protein aggregates, and the corresponding polyphenol and flavonoid content gradually decreased. Whey protein fermentation for 7 h, 7.5 h, and 8 h, and retention in gastric juice for 0.5 h, showed increased protein release compared to the blueberry whey protein mix fermentation gels. The blueberry polyphenols in the mixed system encouraged the formation of protein aggregates that were not easily disrupted, so in the pre-gastric fluid response, the release of soluble proteins was lower than in the whey protein gels. Comparing the fermentation times of the blueberry-lactalbumin, the blueberry-lactalbumin fermented for 7.5 h was lower than that at 7 h and 8 h in the gastric acid response for 1–2 h, presumably because fermentation for 7.5 h was more favourable for the formation of blueberry gels, and the blueberry gel matrix was more stable, harder, and not as easily destroyed by the gastric acid.

Blueberries are considered to be a rich source of phenolic compounds, and polyphenol–protein aggregates are a source of anthocyanins. A number of studies have shown that polyphenol content is related to fermentation time, with higher polyphenol content at 0.5–1 h and 7.5 h of fermentation, suggesting that 7.5 h of fermentation is more conducive to polyphenol storage. As the protein polyphenol aggregates break down at 0.5–1 h, the polyphenol content increases, as does the protein content. It was clear from the literature that at the point of highest protein content, the polyphenols were protected by the thin protein layer in the blueberry extract, so at 1.5 h of the reaction, the protein content was highest and the polyphenol content decreased. After 1.5 h, as the free protein decreased, combined with the gel being broken up, and the polyphenol content began to increase [19].

In simulated gastric juice experiments, the flavonoid release is typically limited and varies very little. The flavonoid content increased slowly at 0–1.5 h and decreased at 1.5 h–2 h. The basic structure of flavonoids is the C6-C3-C6 carbon backbone, which consists of two phenyl rings (A and B) and an oxygen-containing heterocycle (C). Flavonoids have a high number of phenolic hydroxyl groups, C2 = C3 double bonds, and dehydroglucoside structures, which are strongly associated with protein binding. Regardless of the pH of the sample, a higher protein concentration enhanced the hardness of the sample and was detrimental to the release of flavonoids [20]. Therefore, as the protein content increased, the flavonoids increased slowly. At 1.5–2 h, the study showed that the higher the flavonoid content, the greater the ability to scavenge hydroxyl radicals. However, flavonoids and proteins relied mainly on non-covalent binding (e.g., water bonding and hydrophobic interactions). If they bind to the hydrophobic sites of the proteins, the structure of the protein is altered and the flavonoid content is reduced, ultimately weakening its ability to scavenge hydroxyl radicals. Therefore, free proteins and flavonoids were lower. The flavonoids released at 0–1.5 h with fermentation at 7.5 h were lowest, probably because at 7.5 h (Figure 2) the gel was at its hardest, with minimal gastric acid action, and the flavonoids were protected and released only in small amounts.

### 2.5. Rheological Measurements

**Figure 5:**
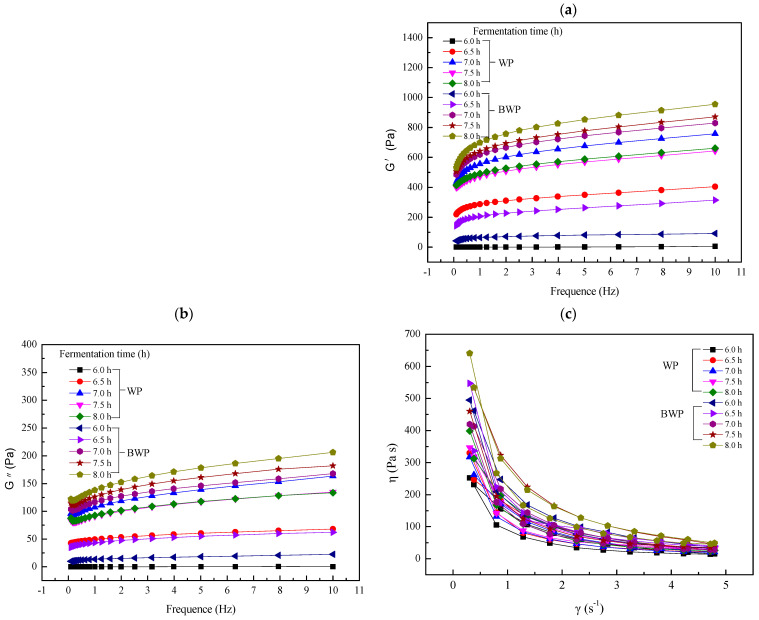
(**a**) Frequency sweep of the storage modulus (G′) and (**b**) loss modulus (G″) of whey protein (WP) and whey protein mixture blueberry juice (BWP) gels formed by *L. plantarum 67* and *L. paracasei* W125 fermentation for different periods of time. (**c**) Apparent viscosity change of whey protein gels compared to blueberry juice gels.

To explain the macroscopic characteristics of different fermented whey and blueberry juice fermented gels, the storage modulus (G′) and loss modulus (G″) were further evaluated. As shown in Figure 5a, the storage modulus (G′) of whey and blueberry fermented gels was higher than the loss modulus (G″), indicating that the gel network was not disrupted. As shown in Figure 5b, with increased fermentation time, because the protein molecule unfolds into the network structure, the network structure was accelerated, which led to an increasing storage modulus (G″) and loss modulus (G″). The whey protein of the 67 + W125 system and the blueberry and whey mixture fermented system had a higher storage modulus (G′) after fermentation for 7.5 h. The blueberry juice addition significantly improved the elasticity and viscosity of the samples. In addition, the system with added blueberry had a higher G′ than the whey protein gel system after 7.5 h of fermentation. This indicated that the addition of blueberry juice enhanced the hardness of the fermented gels, which had a much higher mechanical strength than those prepared with whey alone [21]. The storage modulus (G′) value was increased with fermentation time for both the whey protein and blueberry juice mixtures. A long fermentation time also increased the hardness of the gels. During the fermentation of whey protein, the spatial structure of the proteins change; chemical bonds break, polypeptide chains unfold, and protein polypeptides or new intermolecular or intramolecular chemical bonds are formed, interacting in covalent or non-covalent forms, such as by van der Waals bonds, hydrophobic interactions, and hydrogen bonds. Blueberry addition could initiate the interaction between whey protein and polyphenols in blueberry juice by non-covalent binding (e.g., hydrobonding and hydrophobic interactions) [9]. Furthermore, the G′ and G″ of whey protein and blueberry juice fermented gels after 6.5 h was not changed significantly, indicating that the elastic modulus dominated the whey and blueberry juice gel system, in which the network structure of the gels was not significantly weakened [22]. Therefore, the blueberry and whey protein mixed system was generally larger in terms of G′ and G″ than the whey protein system, possibly because the formation of hydrogen bonds between blueberry and whey proteins results in an increased storage and loss modulus [4]. The value of the storage modulus (G′) and loss modulus (G″) was very low, near to zero, for whey protein fermented gels and whey protein mixed blueberry juice fermented gels at 6.0, indicating the structure of the gels was weak. The gels become hard at fermentation for 6.5 h, as shown in Figure 5a. In Figure 5c, the apparent viscosity can also be seen as increasing with the fermentation time increase. The apparent viscosity was at the minimum at fermentation for 6.0 h, with an increase in the shear rate. The apparent viscosity value was 252.2 at 8.0 h of fermentation. When the apparent viscosity reached the maximum, the value was 398.6. The blueberry and whey concentrated protein mixed system had the higher apparent viscosity, at 241.9 higher than the maximum apparent viscosity value of the concentrated whey protein gel system.

### 2.6. Texture Analysis of Whey Protein Blueberry Gel Formation at Different Fermentation Times

As shown in Table 2, the texture changes of different samples varied at different fermentation times. The hardness of whey protein gels and whey mixed blueberry juice gels increased as fermentation time increased from 6 h to 7.5 h. Then, the hardness of the gels decreased at 8 h. The adhesiveness of the gels was increased with increased fermentation time. The integrity of the pellets is determined by their cohesiveness. The term “cohesion” was first defined in food science as the strength of the internal bonds that make up the body of a food. Increased cohesion leads to decreased flow capacity. A low cohesive pellet that breaks apart easily and forms a residue is not suitable for swallowing. Appropriately cohesive food in the swallowing-stretch stage is not easy to break and can be swallowed more smoothly. The cohesiveness of pellets is therefore essential for swallowing. Elasticity is the ability of the sample to deform and return to its initial state after being subjected to external forces [23]. The cohesiveness was not significantly different between whey protein gels and whey mixed blueberry juice gels with increased fermentation time. The elasticity of the gels was significantly increased at fermentation for 8 h compared to 6 h. This indicated that gels fermented for 8 h had an improved ability to recover their initial state, though their hardness was decreased. The chewiness of the whey protein fermented gels was increased as fermentation time increased, which indicated that a more compact structure was formed. However, the chewiness of whey protein mixed blueberry juice was decreased at fermentation between 6 and 7 h, before it increased significantly at 7.5 h and decreased at 8 h. The chewiness of whey protein mixed blueberry juice was higher than that of whey protein fermented gels at 7.5 h and 8 h. This was associated with the intermolecular hydrogen bonding and lack of covalent bonding; intermolecular hydrogen bonds exist between blueberry juice and whey protein following fermentation for 7.5 h and 8 h [24]. The maximum rupture force and hardness were greater than the maximum rupture force and hardness at the beginning of the gel formation process. The blueberry and whey concentrated protein mixture system seeded with 67 + W125 reached the maximum rupture force, hardness, and adhesive viscosity at 8 h of fermentation. Adherence and elasticity were maximized by fermentation for 8 h.

### 2.7. Monitoring of Live Bacteria during Storage of Whey Protein in Blueberry Gels

**Figure 6:**
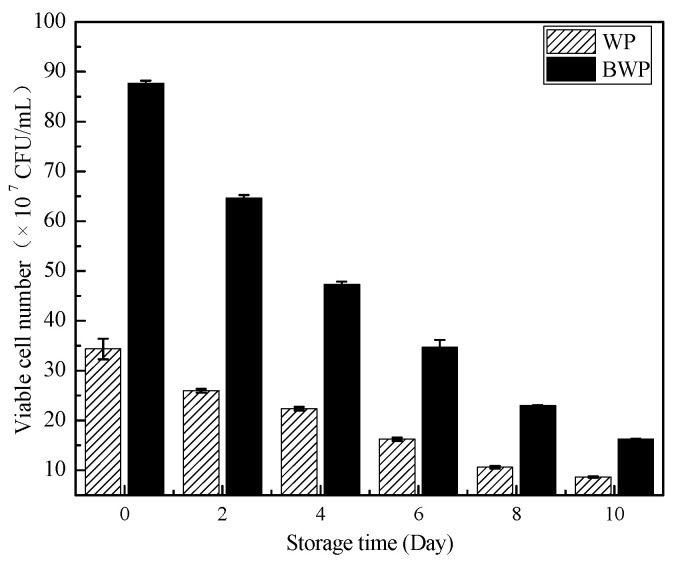
The viable cell number of whey protein fermented gels (WP) and whey protein mixture blueberry juice gels (BWP) formed by *L. plantarum 67* and *L. paracasei* W125 during storage.

As shown in Figure 6, lactic acid bacteria still existed in the fermented gels stored for 10 d, though the viable cell number decreased. The gels of the whey protein and blueberry juice fermented samples had a higher viable cell number than the whey protein fermented gels. The initial viable cell number (0 d) was higher for whey protein and blueberry juice gels. This was due to the structure of the gels; the whey protein and blueberry juice fermented gels had more small pores, giving them a honeycomb structure, compared to the whey protein fermented gels, as shown in Figure 2. A honeycomb structure is beneficial to the adsorption of lactic acid bacteria and increases the adsorption capacity of bacteria. The viable cell number was 10^7^–10^8^ CFU/mL for whey protein and blueberry juice fermented gels after storage for 10 d. However, the viable cell number was lower than 10^8^ for whey fermented gels after storage for 10 d. Therefore, blueberry juice addition to whey protein not only helps with the formation of a honeycomb structure but could also increase the viable cell number during storage.

## 3. Conclusions

At 7 h of fermentation, gels were formed and observed under an electron microscope. The network structure formed between the gels became tighter, denser, and more three-dimensional as the fermentation time increased, but there was no significant change in appearance or colour. At the same time, as the structure became denser, the water-holding capacity became progressively greater with increasing fermentation time. The longer the fermentation time, the tighter the network structure and the fewer water molecules that could enter the network structure. The water molecules and proteases entering the network structure first increased and then levelled out under the digestion culture of SGF. This explained why, after 1 h of digestion incubation in SGF, the gels showed the greatest swelling and a higher release of polyphenols and flavonoids. When texture was considered, the whey concentrate was significantly harder at 7 h of fermentation, and the blueberry and whey concentrate mixed gel system was hardest at 8 h of fermentation. Finally, based on rheological measurements of the gels, the storage and loss moduli of the gels increased progressively with frequency, and were greater for the blueberry and whey protein concentrate blend gels. The apparent viscosity decreased with increasing shear and was greater for the blueberry and whey protein concentrate blend gels, consistent with the texture measurements. Various measurements showed that the gels formed by the whey protein concentrate were of better quality.

## 4. Materials and Methods

### 4.1. Materials

The 80% whey protein concentrate (WPC80) was from New Zealand. Blueberry samples were obtained from the Heihe region of China. *L. plantarum* 67 and *L. paracasei* W125 were from Yangzhou University, Yangzhou, China.

### 4.2. Experimental Preparation

First, *L. plantarum* 67 and *L. paracasei* W125 were grown to the second generation. Blueberry juice (16.67 mL) and sugar (6 g) were dissolved in water to 100 mL. The first sample was the blueberry juice (16.67 mL), sugar (6 g), and whey protein concentrate (6 g) with distilled water to 100 mL. The other sample was sugar (6 g) and whey protein concentrate (6 g) with distilled water to 100 mL. The two samples were hydrated at 35 °C for 20 min to fully dissolve, and then were mixed. The pH value was adjusted to 6.5 with sodium hydroxide, and pasteurization was performed at 95 °C for 5 min. *Lactiplantibacillus plantarum* (1.5 mL/100 mL) and *L. paracasei* (1.5 mL/100 mL) were cultured and fermented at 37 °C for 6 h, 6.5 h, 7 h, 7.5 h, and 8 h, respectively, giving 10 samples total [15]. The sample of whey protein + *L. plantarum* 67 + *L. paracasei* W125 is abbreviated as WP, and the sample of whey protein blueberry + *L. plantarum* 67 + *L. paracasei* W125 is abbreviated as BWP.

### 4.3. Surface Structure Observation

Gels with different fermentation times were selected (in principle, there was a visual gap), and the status of the blueberry juice and whey concentrated protein mixed liquid at 6 h, 6.5 h, 7 h, 7.5 h, and 8 h, and during the gel formation of the whey concentrated protein system, were observed using a camera (Canon, Tokyo, Japan). Gel samples were freeze-dried for 12 h for SEM (Carl Zeiss, Oberkoche, Germany). Then, the different fermented gels were also observed using a scanning electron microscope at 30×, 200×, and 3000× magnification and at an acceleration voltage of 5.0 kV [25].

### 4.4. Colour Measurement of Each Sample at Different Fermentation Times

A certain number of gel samples were selected, and the L, a, and b values of the gels were determined at room temperature by a chromatometer (KONICA MINOLTA, Tokyo, Japan), where L was the brightness of the sample, a was the red (+) and green (−) value of colour, and b was the yellow (+) and blue (−) value of colour. Each group of samples gave three parallel readings [26].

### 4.5. Texture of Whey Protein or Blueberry Gel Formation at Different Fermentation Times

Samples with different fermentation times were selected and the texture of each sample was measured using a plasgraph, which penetrated the sample with a cylindrical stainless steel probe (7.5 mm diameter) at 60 mm/min, to up to 60% of the gel height. The probe was 75 mm. The protostomer automatically generated hardness, viscosity, cohesion, elasticity, and masticability data, and each sample was measured in parallel three times [27].

### 4.6. Water Holding Capacity of Whey Protein or Blueberry Gels

The 8 g gel samples with different fermentation times at pH 6.5 were put into centrifuge tubes, and the total mass was weighed at 25 °C, followed by 1500 rpm for 15 min, drained and weighed, and finally calculated according to the following calculation formula [17]:

WHC (%) = (m_2_ − m_0_)/(m_1_ − m_0_) ∗ 100% (1)

m_0_: the mass of the centrifuge tube (g). m_1_: the mass before centrifuging (g). m_2_: the mass of the gel after centrifuging (g).

### 4.7. Swelling and Protein Release following In Vitro Digestion

A total of 37% of 7.0 mL of hydrochloric acid and 2.0 g of sodium chloride (NaCl) were dissolved in 1000 mL of deionized water, with a pH of 1.2, to make enzyme-free simulated gastric juice. The prepared gel samples were removed, and the gels were dried with absorbent paper then weighed, then were immersed in enzyme-free simulated gastric juice (SGF) at 37 °C in an incubator (Jing Hong, China). Regular samples were taken out, gently wiped and reweighed, and the following equation was used to calculate the swelling rate [17]:

Swelling = [(W_t_ − W_0_)/W_0_] ∗ 100% (2)

W_0_: the initial gel weight. W_t_: the gel weight at different digestion times.

GF samples combined with pepsin (3.2 g/L) were shaken at 100 rpm at 37 °C for up to 2 h. The gel samples were centrifuged, and the whey protein in the supernatant was measured at 280 nm to determine the released protein.

Degradation (%) = (released protein of gel/total protein in gel) ∗ 100%(3)

The protein released was the amount of protein released into the SGF, and the total protein was the amount of protein in the gel matrix.

### 4.8. Release of Total Phenolic Content (TPC) following In Vitro Digestion

The Folin–Ciocalteu method was used to examine the TPC of the samples [28].

### 4.9. Release of Anthocyanin Concentration (TAC) following In Vitro Digestion

The gels were mixed with 9 mL potassium chloride (KCl) buffer (0.025 mol/L, pH 1.0) and sodium acetate (CH_3_COONa) buffer (0.4 mol/L, pH 4.5) in two centrifuge tubes. The mixture was incubated for 15–30 min, and the absorbance was measured at 520 nm and 700 nm, as previously reported [29].

### 4.10. Rheological Measurements

Rheological measurements were taken with a Malvin rheometer (Malvin, Malvern UK). Gel samples for each fermentation time were investigated. The apparent viscosity parameters were assessed at a temperature of 20 °C and a shear rate of 30 r/min; we set the parameters of the storage modulus and loss modulus, and the shear rate increased from 0 r/min to 300 r/min. The same but negative acceleration decreased until the shear rate decreased to 0. The linear viscoelastic region was tested by stress scanning at a fixed frequency (1 Hz). The energy storage modulus (G′) and loss modulus (G″) were evaluated under strain in the linear range (0.01~100%) [6,30].

### 4.11. Monitoring of Live Bacteria during Storage of Whey Protein in Blueberry Gels

MRS solid medium and 0.9% saline were configured and sterilized at 121 °C for 15 min. The concentrated whey protein gel and blueberry whey protein gel were incubated by shaking, and the resulting sample solution was diluted to 10^−5^, 10^−6^, and 10^−7^ with MRS medium. The MRS medium was adjusted to pH 7.0. The MRS dish was placed in a 37 °C incubator for 48 h to count the number of colonies [31]. MRS medium: peptone 10.0 g/L, beef extract 10.0 g/L, yeast extract 5.0 g/L, glucose 20.0 g/L, lactose 20.0 g/L, dipotassium phosphate 2.0 g/L, triammonium citrate 2.0 g/L, sodium acetate 5.0 g/L, magnesium sulfate heptahydrate 0.2 g/L, manganese sulfate tetrahydrate 0.04 g/L, T80 1.0 g/L; pH adjusted to 6.8 with 6 mol/L hydrochloric acid, and deionized water added to 1.0 L. Sterilized at 121 °C for 15 min, and cooled for use, before 1.5% AGAR powder was added in solid medium.

## Figures and Tables

**Table 1:** The colour changes of whey protein mixture blueberry juice gels formed by *L. plantarum* 67 and *L. paracasei* W125 at different fermentation times.

Sample (pH 6.5)	Fermentation Time (h)	ΔL	Δa	Δb	ΔE
Whey protein *L. plantarum* 67 and *L. paracasei* W125	6 h	4.167 ± 0.988 ^b^	0.180 ± 0.206 ^b^	2.647 ± 0.988 ^b^	4.968 ± 1.361 ^c^
	6.5 h	7.213 ± 0.859 ^a^	0.450 ± 0.408 ^a^	5.693 ± 0.859 ^a^	9.220 ± 1.211 ^a^
	7 h	5.387 ± 0.596 ^b^	0.033 ± 0.269 ^c^	3.867 ± 0.596 ^b^	6.644 ± 0.834 ^b^
	7.5 h	5.023 ± 1.337 ^b^	0.157 ± 0.239 ^b^	3.503 ± 1.337 ^b^	6.158 ± 1.854 ^b^
	8 h	5.260 ± 0.601 ^b^	0.173 ± 0.272 ^b^	3.740 ± 0.601 ^b^	6.472 ± 0.835 ^b^
Blueberry + Whey protein *L. plantarum* 67 and *L. paracasei* W125	6 h	8.980 ± 0.264 ^b^	3.510 ± 0.553 ^a^	0.210 ± 0.445 ^a^	9.683 ± 0.445 ^b^
	6.5 h	11.02 ± 0.220 ^a^	3.327 ± 0.533 ^a^	0.063 ± 0.258 ^b^	11.57 ± 0.332 ^a^
	7 h	11.41 ± 0.728 ^a^	4.113 ± 0.408 ^a^	−0.807 ± 0.373 ^c^	12.19 ± 0.657 ^a^
	7.5 h	12.23 ± 0.429 ^a^	4.223 ± 0.187 ^a^	0.100 ± 0.274 ^b^	12.95 ± 0.469 ^a^
	8 h	11.95 ± 0.797 ^a^	3.607 ± 0.176 ^a^	−0.617 ± 0.784 ^b^	12.56 ± 0.721 ^a^

The same letter denotes insignificant changes (*p* > 0.05), different letters denote significant changes (*p* < 0.05). The difference was assessed by intra-group comparison of whey gels and whey protein mixture blueberry juice gels.

**Table 2:** Texture changes of whey protein mixture blueberry juice gels formed by *L. plantarum 67* and *L. paracasei* W125 following fermentation for different periods of time.

Sample (pH 6.5)	Fermentation Time (h)	Rupture Strength (N)	Hardness (N)	Maximum Adhesion (N·M^−2^)	Adhesiveness (%)	Cohesiveness (%)	Elasticity	Glueyness	Chewiness
Whey protein *L. plantarum* 67 and *L. paracasei* W125	6.0 h	0.619 ± 0.084 ^b^	0.674 ± 0.049 ^c^	−0.086 ± 0.012 ^b^	1.023 ± 0.101 ^b^	0.490 ± 0.000 ^a^	16.890 ± 0.072 ^b^	0.322 ± 0.026 ^b^	5.440 ± 0.435 ^b^
	6.5 h	0.707 ± 0.052 ^b^	1.094 ± 0.503 ^b^	−0.112 ± 0.014 ^b^	1.218 ± 0.205 ^b^	0.493 ± 0.012 ^a^	15.933 ± 0.409 ^b^	0.462 ± 0.091 ^b^	7.353 ± 1.384 ^b^
	7.0 h	1.289 ± 0.535 ^a^	1.850 ± 0.111 ^a^	−0.094 ± 0.019 ^b^	1.248 ± 0.290 ^b^	0.485 ± 0.007 ^a^	17.312 ± 0.160 ^b^	0.682 ± 0.064 ^a^	11.80 ± 1.088 ^a^
	7.5 h	0.929 ± 0.429 ^b^	1.216 ± 0.274 ^b^	−0.119 ± 0.027 ^b^	1.434 ± 0.647 ^b^	0.455 ± 0.037 ^a^	16.033 ± 4.397 ^b^	0.620 ± 0.064 ^a^	10.14 ± 3.370 ^a^
	8.0 h	0.703 ± 0.156 ^b^	1.185 ± 0.077 ^b^	−0.150 ± 0.006 ^a^	1.980 ± 0.073 ^a^	0.418 ± 0.062 ^a^	19.380 ± 0.125 ^a^	0.646 ± 0.072 ^a^	12.51 ± 1.365 ^a^
Blueberry + Whey protein *L. plantarum* 67 and *L. paracasei* W125	6.0 h	0.975 ± 0.037 ^b^	1.096 ± 0.171 ^b^	−0.140 ± 0.030 ^b^	1.638 ± 0.388 ^c^	0.480 ± 0.034 ^a^	17.277 ± 0.146 ^b^	0.504 ± 0.086 ^b^	8.713 ± 1.533 ^b^
	6.5 h	0.707 ± 0.052 ^b^	1.094 ± 0.503 ^b^	−0.112 ± 0.014 ^b^	1.218 ± 0.205 ^d^	0.493 ± 0.012 ^a^	15.933 ± 0.409 ^b^	0.462 ± 0.091 ^b^	7.353 ± 1.384 ^b^
	7.0 h	0.778 ± 0.426 ^b^	1.102 ± 0.110 ^b^	−0.166 ± 0.025 ^b^	1.784 ± 0.241 ^c^	0.495 ± 0.037 ^a^	17.563 ± 0.297 ^b^	0.397 ± 0.106 ^b^	6.960 ± 1.785 ^b^
	7.5 h	1.243 ± 0.303 ^a^	1.692 ± 0.133 ^a^	−0.241 ± 0.045 ^a^	3.267 ± 0.427 ^a^	0.543 ± 0.065 ^a^	19.235 ± 0.202 ^a^	0.785 ± 0.124 ^a^	15.11 ± 2.374 ^a^
	8.0 h	1.329 ± 0.232 ^a^	1.542 ± 0.115 ^a^	−0.197 ± 0.024 ^a^	2.804 ± 0.249 ^b^	0.493 ± 0.031 ^a^	19.710 ± 0.295 ^a^	0.686 ± 0.102 ^a^	13.53 ± 2.180 ^a^

The same letter denotes insignificant differences (*p* > 0.05), while a different letter denotes a significant difference (*p* < 0.05). The difference was assessed by intra-group comparison of whey gels and whey protein mixture blueberry juice gels.

## Data Availability

The datasets are available from the corresponding author on reasonable request.
